# Applying functional near-infrared spectroscopy and eye-tracking in a naturalistic educational environment to investigate physiological aspects that underlie the cognitive effort of children during mental rotation tests

**DOI:** 10.3389/fnhum.2022.889806

**Published:** 2022-08-12

**Authors:** Raimundo da Silva Soares, Amanda Yumi Ambriola Oku, Cândida S. F. Barreto, João Ricardo Sato

**Affiliations:** ^1^Center for Mathematics, Computation and Cognition, Universidade Federal do ABC, São Bernardo do Campo, Brazil; ^2^Graduate Program in Neuroscience and Cognition, Federal University of ABC, São Bernardo do Campo, Brazil; ^3^South African National Research Foundation Research Chair, Faculty of Education, University of Johannesburg, Johannesburg, South Africa

**Keywords:** spatial cognition, cognitive effort, pupillometry, fNIRS, naturalistic experimentation, educational research

## Abstract

Spatial cognition is related to academic achievement in science, technology, engineering, and mathematics (STEM) domains. Neuroimaging studies suggest that brain regions’ activation might be related to the general cognitive effort while solving mental rotation tasks (MRT). In this study, we evaluate the mental effort of children performing MRT tasks by measuring brain activation and pupil dilation. We use functional near-infrared spectroscopy (fNIRS) concurrently to collect brain hemodynamic responses from children’s prefrontal cortex (PFC) and an Eye-tracking system to measure pupil dilation during MRT. Thirty-two healthy students aged 9–11 participated in this experiment. Behavioral measurements such as task performance on geometry problem-solving tests and MRT scores were also collected. The results were significant positive correlations between the children’s MRT and geometry problem-solving test scores. There are also significant positive correlations between dorsolateral PFC (dlPFC) hemodynamic signals and visuospatial task performances (MRT and geometry problem-solving scores). Moreover, we found significant activation in the amplitude of deoxy-Hb variation on the dlPFC and that pupil diameter increased during the MRT, suggesting that both physiological responses are related to mental effort processes during the visuospatial task. Our findings indicate that children with more mental effort under the task performed better. The multimodal approach to monitoring students’ mental effort can be of great interest in providing objective feedback on cognitive resource conditions and advancing our comprehension of the neural mechanisms that underlie cognitive effort. Hence, the ability to detect two distinct mental states of rest or activation of children during the MRT could eventually lead to an application for investigating the visuospatial skills of young students using naturalistic educational paradigms.

## Introduction

Spatial cognition is usually described as “the capacity to generate, retain, retrieve, and transform well-structured visual images in mind” (Lohman, 1994). This ability to understand and manipulate the objects’ spatial characteristics has been associated with academic achievement in science, technology, engineering, and mathematics (STEM) domains (Wai et al., 2009; Khine, 2016). Psychology research has often measured spatial cognition via visuospatial tasks, including the mental rotation task (MRT) paradigm (Shepard and Metzler, 1971, 1988; Bruce and Hawes, 2015; Sladky et al., 2016). MRT requires the subject to determine whether pairs of figures exhibited are the same or mirror images. To accomplish this task, the subject creates a mental image and rotates it into a different orientation, which is related to spatial reasoning, visuospatial ability, and resembles everyday situations that require spatial orientation and the capacity to manipulate objects mentally (Guillot et al., 2012).

In cognitive neuroscience, the MRT has been applied to different ages and neural development stages. Studies with adolescents and adults indicate a relationship between MRT and mathematical achievement (Kyttälä and Lehto, 2008; Tolar et al., 2009; Wei et al., 2012). Visuospatial cognition studies on children highlight the relevance of spatial ability in many scientific domains and support intervention to develop spatial skills (Webb et al., 2007; Tzuriel and Egozi, 2010). However, there are very few studies that investigate the brain activity of children during MRT (Roberts and Bell, 2000; Wu et al., 2020). Indeed, many studies examine the neural substrate associated with MRT and its relationship to adults’ learning process (Jordan et al., 2001; Harris and Miniussi, 2003; Zacks et al., 2003; Zacks, 2008). Neuroimaging studies have shown that some brain regions, including the superior parietal [Brodmann’s area (BA) 40], premotor cortex (BA 6), and the frontal cortices [dorsolateral prefrontal cortex (dlPFC) BAs 9 and 46; ventrolateral prefrontal cortex (vlPFC) BA 44], are involved with bilateral activation regions during the MRT (Cohen et al., 1996; Hugdahl et al., 2006; Zacks, 2008; Wu et al., 2020). Other studies found that the modulation of activity in the frontal cortex reflects the participant’s cognitive effort, i.e., mental resources required under cognitive demands (Prat et al., 2007; Ayaz et al., 2010, 2012a,b; Fishburn et al., 2014; Causse et al., 2017). For example, Causse et al. (2017) demonstrated that the functional near-infrared spectroscopy (fNIRS) was sensitive to task difficulty variations. Also, the prefrontal activation intensity sheds insight on the level of mental effort, indicating the amount of cognitive workload required by demanding tasks. Importantly, there are age-related differences in cognitively demands of mental processing and performance (McCabe et al., 2010). Older adults tend to express a higher effortful reaction and lower performance on cognitively demanding activity (Hess and Ennis, 2012; Westbrook et al., 2013), suggesting that poor performance could be a sign of depletion or reduced cognitive resources. It seems that greater levels of mental effort are related to lower performance (Hess and Ennis, 2012). Yet, little is known about how schoolchildren’s cognitive effort relates to students’ performance (Chevalier, 2018), and physiological measurements could be helpful to address this matter.

Eye-tracking (ET) is another reliable technological tool for research in the educational context. It provides a non-invasive and real-time measurement of pupil sizes from subjects during cognitive tasks (Lum et al., 2017; McGarrigle et al., 2017). Pupillometry studies indicate that pupil diameter changes in response to mental activity and increases with task difficulty in children (Boersma et al., 1970; Karatekin, 2004; Karatekin et al., 2007; Chatham et al., 2009; Johnson et al., 2014; Rojas-Líbano et al., 2019) and adults (Hess and Polt, 1964; Beatty and Kahneman, 1966; Granholm et al., 1996). Pupillometry measures during cognitive demanding mental arithmetic tasks suggest that pupil dilation may indicate mental activity, which includes mental arithmetic tasks (Hess and Polt, 1964; Ahern and Beatty, 1979; Jainta and Baccino, 2010; Klingner et al., 2011) and spatial ability tasks (Buckley et al., 2018; Campbell et al., 2018; Bauer et al., 2021a,b). For example, Toth and Campbell (2019) showed that the cognitive demand of the MRT elicited increases in participants’ pupil diameters (Toth and Campbell, 2019). It is well known that the noradrenergic system has been implicated in mental effort and pupil dilation. However, the precise mechanism related to PFC activity and fluctuations in pupil size is still not fully understood (Bradley et al., 2008; Mathot, 2018).

There is growing recognition that fNIRS is moderately tolerant to motion artifacts and is a portable, cost-effective device (Ferrari and Quaresima, 2012; Curtin and Ayaz, 2018). All these together make fNIRS a suitable tool to use in conjunction with an eye-tracker device for monitoring the brain activity of children in a naturalistic setting such as realistic educational situations. Together, pupillometry and neuroimaging techniques are valuable for integrating mental effort signs and shedding light on the neural mechanisms that underlie cognitive load (Hosseini et al., 2017; Ís̨bilir et al., 2019). Although previous studies have investigated the cognitive effort’s neural mechanism in adults, little is known about children’s brain activity under visuospatial cognitive demands. Considering the spatial cognition relevance to school achievement (Giofrè et al., 2013), physiological measurements of students’ mental effort during visuospatial tasks will make it possible to advance the understanding of cognitive aspects related to academic performance, specially geometry problem-solving development. We believe that traditional cognitive load measures could benefit from ET-fNIRS-based mental workload detection by combining additional information sources related to the mental effort of children during challenging tasks. Such a multimodal approach has the potential to contribute to a better understanding of young students’ cognitive resources that underlie the learning process.

Multimodal applications can enhance physiological response detection from different modalities, which enable more accurate and robust measurements of cognitive states (Siegle et al., 2003; Shin et al., 2018), including mental effort (Laeng et al., 2012; Rozado and Andreas, 2015). Therefore, we propose the ET-fNIRS fusion as a promising framework for future investigations in educational research. Here, we sought to shed light on physiological responses related to children’s cognitive resources during visuospatial tasks. More specifically, we wanted to improve our understanding of the relationship between pupil dilatation and hemodynamic PFC activity during a visuospatial task in children. Therefore, we use a multimodal approach by applying fNIRS and Eye-tracking simultaneously to investigate primary school children’s mental effort expressed by brain hemodynamics changes and pupil size during the MRT. Based on the research cited above, we hypothesized that the higher bilateral PFC activity and pupil dilatation might be associated with higher mental effort during the spatial task and, consequently, lower children’s performance (i.e., visuospatial scores).

## Materials and methods

### Participants

The local Ethics Committee of UFABC approved all aspects of our experiment. The experiment was performed following all relevant guidelines and federal regulations. All subjects participated voluntarily and without any financial compensation. Thirty-two healthy students (fifteen boys and sixteen girls, 9–11 years old) from the 5th grade of a Brazilian elementary public school participated in this study. Parents/legal guardians of the young children provided written informed consent and were informed verbally of the purpose of the study and the safety of the experiments. All participants had normal or corrected to normal vision, and did not report present or previous neurological or psychiatric conditions.

### Eye-tracking data acquisition

The pupil diameter was recorded using an ASL Mobile Eye-5 (Applied Sciences Laboratory, Bedford, MA, United States) with a capture rate of 60 Hz. The glasses were mounted with one small camera over the right eye. The ASL Mobil Eye-5 auto-calibrates to lighting conditions in the environment and compensates for head movements.

### Functional near-infrared spectroscopy data acquisition

**FIGURE 1:**
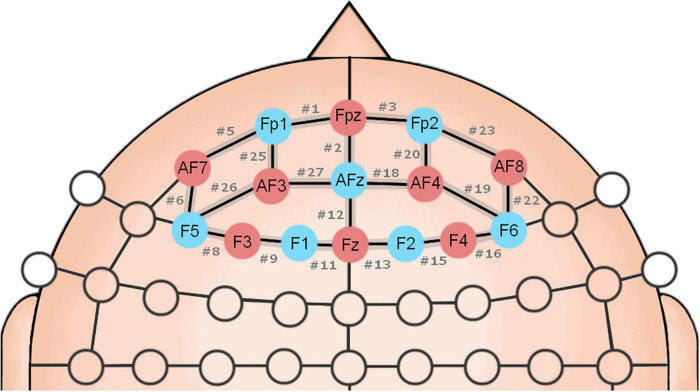
Schematic representation of the fNIRS optodes positioned over the scalp. The position of the optodes follows the universal configuration of the 10-10 EEG international system. Channels are composed of neighboring optodes represented by the black edges, sources in red (S1–S8) corresponding to F3, AF7, AF3, Fz, Fpz, AF4, AF8, F4, and detectors in blue (D1–D7) matching F5, F1, Fp1, AFz, F2, Fp2, F6. # channel.

The hemodynamic signals were recorded using the continuous-wave NIRSport System (NIRx Medical Technologies, Glen Head, NY, United States). The cap montage was an array of eight pairs of optodes (sources and detectors), resulting in 28 channels (twenty long-range channels and eight short-distance channels) placed bilaterally over the prefrontal cortex (Figure 1), including the brain regions of the premotor cortex (BA 6), and dlPFC (BAs 9, 10, and 46; Koessler et al., 2009) (see Supplementary material 1 and Supplementary Figure 1 for more details). Source and detector distance were 30 mm with optodes positioned on the measuring cap regarding the 10–10 international system (Jurcak et al., 2007). The illumination sources emitted two wavelengths of near-infrared light (760 and 850 nm), and the signals were recorded at a sampling rate of 7.91 Hz. A dark cloth was placed over the measuring cap to reduce noise from the external light. We used the software NIRStar 15.2 (NIRx Medical Technologies, Glen Head, NY, United States) to record the brain activity data and to evaluate the signal quality of the channels.

### Geometry problem-solving test

To assess the geometry problem-solving performance, we selected the questions from the Brazilian Basic Education Assessment System (SAEB). It is a national test that assesses the mathematical and language skill levels of Brazilian students from the first and last year of elementary school, 5th and 9th grades, respectively (Carnoy et al., 2015). We selected and printed eleven geometry multiple-choice problems from the SAEB test public database (SAEB, 2011). The test was designed to measure the general geometry problem-solving skills of 5th graders, and we adapted the questions in such a way that the students did not know the results beforehand.

### Mental rotations test

**FIGURE 2:**
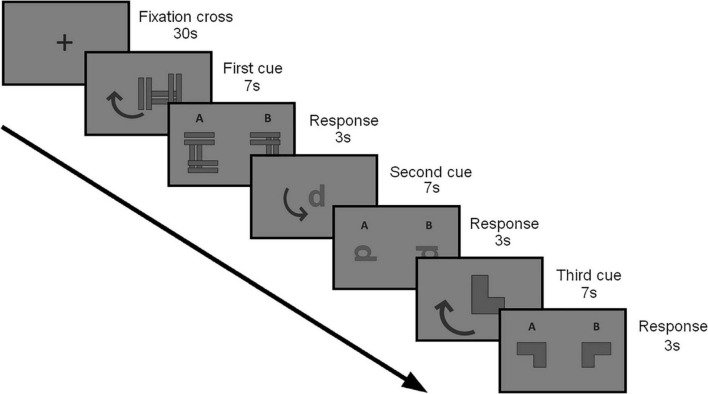
Schematic diagram of the experimental procedure. Fixation cross (the 30 s) was followed by the first cue (7 s) as one gray abstract figure placed on the center of the slide with a gray arrow indicating the left or right direction of rotation. A stimulus “response” was then presented (3 s) as a pair of gray abstract figures identified as “A” and “B” with a similar form, but different orientations at 90° according to the cue (to the right or left visual field) so the participants could answer verbally. The child had 30 s to rest, 7 s to imagine the rotated geometric figure, and 3 s to choose the alternative, three times per block, yielding 30 s of stimuli.

The MRT was adapted for the 9–11 years old children. The task consisted of 70 slides with images in two dimensions following the geometrical patterns (abstract figures). There were two kinds of abstract figures (Figure 2) in the stimuli session: (1) one gray abstract figure placed on the center of the slide with a gray arrow indicating the left or right direction of rotation to the next slide, and (2) a pair of gray abstract figures identified as “A” and “B” with a similar form, but different orientations. Only one of the options was a 90° rotation of the figure presented before. The task requires children to imagine the movement of rotation in the first slide to decide, in the next slide, which of the choices, “A” or “B,” match the stimuli presented before. There were ten trials with three slides of stimuli, three slides of alternatives, and one slide of the control condition in which subjects were instructed to fixate on the cross until the onset of the stimuli. The child had 30 s to rest, 7 s to imagine the rotated geometric figure, and 3 s to choose the alternative, three times per block, yielding 30 s of stimuli. All the sessions had the same level of difficulty to counterbalance the different phases of the task. We used the software NIRStim (NIRx Medical Technologies, Glen Head, NY, United States) to present the visual stimuli and collect the fNIRS data.

### Experimental design

The MRT was performed in the students’ usual place at school. Each child participated in the task individually in a session that lasted approximately 30 min. First, students were randomly called and asked to answer eleven geometry questions from the Brazilian basic education assessment system (SAEB). After solving all the eleven mathematical problems with multiple choices printed on paper, participants were assigned to another room fitted with the fNIRS and Eye-tracker. Two experimenters controlled the equipment and observed the child during the task. Participants were instructed about the experiment and asked to answer a practice trial of MRT to make sure that they understood the task. If the child made a mistake, the experimenter would repeat the trial. After the instruction trials, experimenters set up the fNIRS and eye-tracker. Students were then told that they would next play on the computer screen the same kind of game as in the instruction trials of MRT. The stimuli were presented, followed by the choice “A” and “B,” in which students answered verbally. Participants’ answers were collected during the experiment for further analysis.

### Eye-tracking data analysis

Five children were excluded from the sample due to excessive missing pupil data on the MRT (bad frames), yielding data from twenty-six children. The data were inspected for artifacts such as eye blinks or undetected signals during the task. The high-frequency noise also was also considered an eye tracker artifact. Therefore, we removed the noise from the data by smoothing it using a moving median (Gao et al., 2007). Then we implemented a computer algorithm to automate the artifact removal process for all subjects. We considered recordings insufficient when less than 40% of data remained after cleaning the data to remove artifacts. Children’s median pupil dilation variation (%) was calculated over each second of the task compared to the previous control condition. Then, we determined the median across the experimental trials. The 10 s interval preceding each stimuli block was treated as the baseline. Finally, a *t*-test was applied to test significant differences in the pupil dilation mean between control and task conditions.

### Functional near-infrared spectroscopy data analysis

The hemodynamic signals were processed and analyzed by the software nirsLAB v2019.04 (NIRx Medical Technologies, Glen Head, NY, United States). The signals were truncated before the first block and after the end of the last block. Then, the modified Beer-Lambert law was applied to convert the optical signals of each wavelength (760 and 850 nm) in concentration changes of oxy-Hb and deoxy-Hb. The general linear model (GLM) method (Huppert, 2016) was applied to analyze changes in both oxy-Hb and deoxy-Hb amplitudes during the MRT. In the individual-level analysis, we obtained the activation beta coefficients for each channel of the participants. Then, we used a group GLM to combine all participants’ beta coefficients yielding the group statistical activation map (*t*-test). The rate of accepted false-positive results was set at 5%. Bonferroni correction was applied for multiple comparisons for 20 long-distance channels (resulting *p*-value threshold: <0.0025). The hemodynamic signals were extracted from the channels that presented significant activation in the group analysis. Finally, we calculated the signal block average among the participants to evaluate the channel’s hemodynamic response. The spatial representation of the brain regions related to each channel was rendered from the BrainNet Viewer toolbox (Xia et al., 2013)^1^.

### Behavior data analysis

We analyzed the children’s performance on the geometry problem-solving test and the MRT by counting the correct answers on tasks. The scores on both visuospatial tasks were correlated by Spearman’s correlation coefficient. We also performed Spearman’s correlation analysis to quantify the potential association between behavioral data with pupillary responses and cortical activation. In all cases, *p* < 0.05 (uncorrected) was considered statistically significant. When the short-distance channels were statistically significant to behavioral data, we considered the long-distance correspondent channel insufficient quality data for correlation analysis.

## Results

### Pupillary response

**FIGURE 3:**
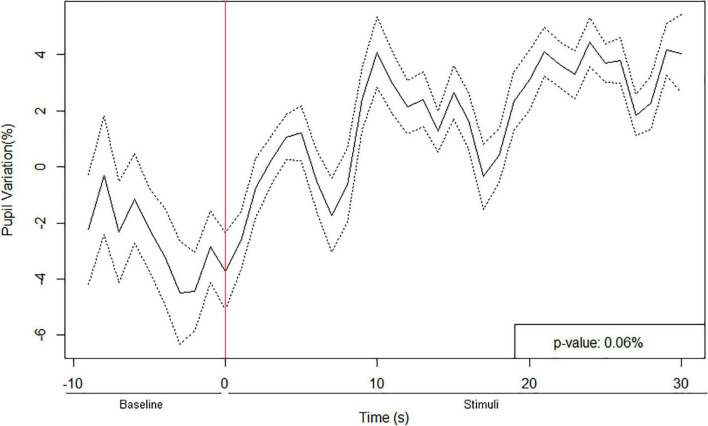
Pupil diameter variation timeline during the MRT. The red line indicates the beginning of the task (stimuli), which had three different figures to rotate per block, yielding 30 s of stimuli (*p* = 0.0006) and 10 s of baseline. The solid line indicates the mean (μ) and the dashed line indicates ± 1 standard error (σ).

The timeline analysis of the pupil size data indicated three distinct periods of pupillary variation (%) from the beginning (0 s) until the end of the MRT (*p* = 0.0006), as shown in Figure 3. First, there was a period without any significant stimuli (10 s of resting state as the baseline), followed by an increase in the children’s pupil size with the stimulus display. The results show a peak of pupil dilation around 10 s after the children imagined the figure. It suggests a pupil dilatation follows a visuospatial task. The responses to the mental rotation stimuli appeared to elicit more pupil dilation than the neutral stimuli. It indicates that it is possible to identify two distinct mental states of rest or activation during the MRT. There is no significant correlation between the mean amplitude of pupil dilation and the number of correct answers, neither in geometric problem-solving nor in the MRT (*p* > 0.05 in all cases).

### Task performance

**FIGURE 4:**
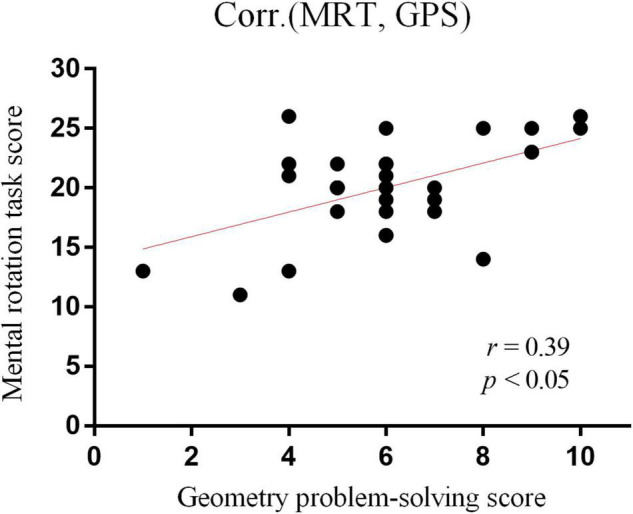
Scatter plot between the number of correct responses on MRT and geometry problem-solving test (scores).

Spearman’s correlation analysis revealed that task performance achieved during the MRT was statistically significantly correlated with the geometry problem-solving performance (*r* = 0.38; *p* = 0.03) (Figure 4).

### Brain-behavior correlation

**FIGURE 5:**
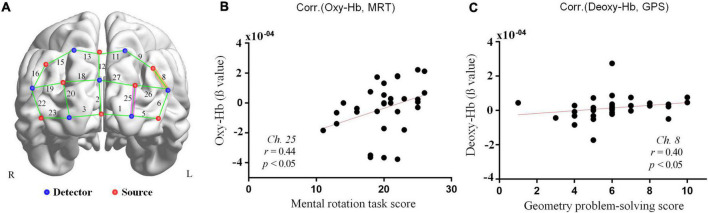
Correlation graphs and fNIRS channels positions. **(A)** 3D-rendered brain template (ICBM152) with left (L) and right (R) hemispheres from BrainNet Viewer toolbox (Xia et al., 2013). The fNIRS channels are positioned across the cortex. The red points represent sources, the blue points are detectors, and the green lines are channels. The purple highlight indicates the channel associated with the correlation between oxy-Hb variation and mental rotation task (MRT). The orange highlight indicates the channel associated with the correlation between Deoxy-Hb variation and geometry problem-solving (GPS). **(B)** Scatter plots between the number of correct responses on MRT and oxy-Hb variation at channel 25 (*r* = 0.43; *p* = 0.01). **(C)** Scatter plots between the number of correct responses on the geometry test and deoxy-hemoglobin (deoxy-Hb) variation at channel 8 (*r* = 0.4; *p* = 0.02).

The Spearman’s correlation analysis revealed correlation between MRT scores and oxy-Hb variation at channel 25 (*r* = 0.43; *p* = 0.01; Figure 5B) and channel 5 (*r* = 0.41; *p* = 0.02), but we considered the channel 5 as insufficient quality data because the corresponding short-distance was also significantly correlated to the task performance (Channel 7; *r* = 0.38; *p* = 0.03; see Supplementary material 3). There is also significant correlation between geometry performance and deoxy-Hb variation at channel 8 (*r* = 0.40; *p* = 0.02; Figure 5C), at short-distance channels 4 (*r* = 0.33; *p* = 0.007), 7 (*r* = 0.41; *p* = 0.02), 24 (*r* = 0.39; *p* = 0.02) and oxy-Hb variation at short-channel 7 (*r* = 0.39; *p* = 0.03; see Supplementary material 3). All other comparisons were not significant. The spatial representation of the brain regions related to each channel is shown in Figure 5A, for the mental rotation tasks scores and the geometry test scores (channels respectively, highlighted in purple and orange). Channel 25 is on the left prefrontal cortex (PFC), and channel 8 is on the left dorsolateral prefrontal cortex (dlPFC).

### Cortical activation

**FIGURE 6:**
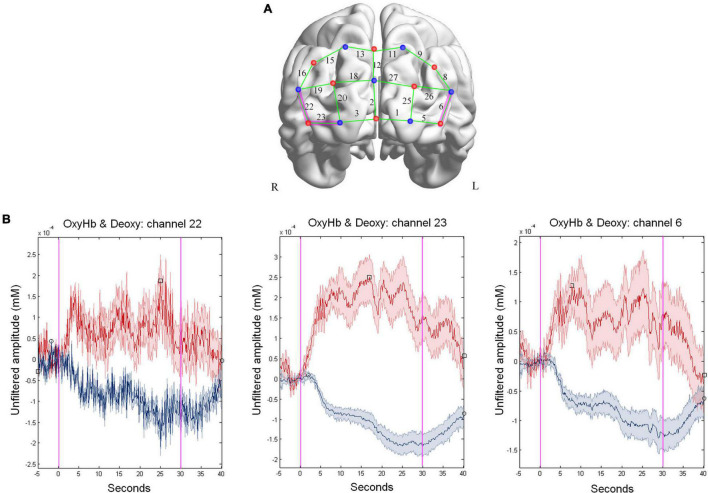
Group level HbO cortical activation map. **(A)** 3D-rendered brain template (ICBM152) with left (L) and right (R) hemispheres from BrainNet Viewer toolbox (Xia et al., 2013). The fNIRS channels are positioned across the cortex. The red points represent sources, the blue- points are detectors, and the green lines are channels. The purple highlight indicates the channel associated with the correlation between deoxy-Hb variation and mental rotation task (MRT). Thresholded SPM *t* image, *p*-value = 0.0025 for deoxy-Hb. **(B)** Block-averaged hemodynamic response (mean ± SD) computed by averaging the oxy-Hb (red) and deoxy-Hb (blue) signals during the MRT for channels 6, 22, and 23.

The results revealed a significant deactivation (Deoxy-Hb) of the left dlPFC (Ch.6; *p*-value corrected = 0.002), and the right dlPFC (Ch.22; *p*-corrected = 0.001; and Ch.23; *p*-value corrected <0.001, see all results in Supplementary material 2). Brain regions represented in Figure 6A show that the channels associated with the correlation between Oxy-Hb variation and MRT are placed on dlPFC. Mean changes in oxy-Hb levels in the other prefrontal regions that we evaluated were not significant. Figure 6B shows the mean group oxy-Hb and deoxy-Hb signal change during the MRT for the channels 6, 22, and 23 (Ch.6, Ch.22, and Ch.23, respectively) indicating that the oxy-Hb concentration increase while the changes in deoxy-Hb were in the opposite direction, as expected.

## Discussion

This study aimed to investigate the 9- to 11-year-old schoolchildren’s cognitive effort expressed by changes in pupil diameter and PFC neural activity under spatial cognitive demand. Our findings suggest that the visuospatial task was able to demand students’ mental effort, which was detectable by the multimodal approach using pupillometry and optical neuroimage. To our knowledge, this is the first study to apply fNIRS and Eye-tracking to assess the cognitive resources required under mental demands through cortical hemodynamic activity and the pupil responses of children during the MRT.

The results indicate a significant positive correlation between the children’s MRT and geometry test scores. There are also significant positive correlations between dlPFC hemodynamic signals and visuospatial task performances (MRT and geometry problem-solving scores). Our results are consistent with dlPFC activity related to higher-level cognitive processing (Miller and Cohen, 2001; Vassena et al., 2014, 2019). Moreover, we found significant activation in the amplitude of deoxy-Hb variation on the dlPFC and that pupil diameter increased during the MRT, suggesting that both physiological responses are related to mental effort processes during the visuospatial task. Our findings indicate that children with more mental effort under the task performed better, which was unexpected. According to the general cognitive load theory (Sweller and Chandler, 1991; Sweller et al., 1998), better problem solvers tend to express less mental effort than novice learners’ performances, as demonstrated in preview studies (Çakır et al., 2011; Shaw et al., 2018). A possible explanation is an age-related difference in our study. Unlike adults, children do not have well-developed visuospatial skills, so they need more activation to solve geometric problems and perform well. Despite the potential to investigate learning and development with eye-tracking, few studies used task-evoked pupil changes analysis on children (Eckstein et al., 2017). So, it is possible to apply pupillometry to elucidate students’ cognitive processes and development.

Spatial cognition improves with age in childhood (Orde, 1996) and declines with age in adulthood (Pak, 2001). Furthermore, studies investigating cognitive efficiency in young and older adults show age-related differences in mental effort and performance (Baltes et al., 1999; Westbrook et al., 2013; Westbrook and Braver, 2015). Older adults tend to express a higher effortful reaction and lower performance on work memory tasks (McCabe et al., 2010; Westbrook et al., 2013) and math problem-solving (Hess and Ennis, 2012). On the other hand, studies with children demonstrated that young children primarily engage the frontal cortex when solving numerical tasks, decreasing by age and differences in brain development (Houdé et al., 2010; Artemenko et al., 2018; Soltanlou et al., 2018a,b). In this sense, we believe that adults would have different findings from children because of neural development. Such information has important implications for understanding students’ spatial cognition and developing an intuitive understanding of fundamental geometric concepts.

A significant positive correlation was observed between MRT and geometry problem-solving performance. It indicates that children with good performance in figure rotation also present better performances in our geometry test based on the Brazilian mathematics assessment. It corroborates that our visuospatial task requires mental rotation skills associated with geometry problem-solving abilities. Geometry is a fundamental approach to interpreting the physical environment and everyday situations (Hwang et al., 2009). It contains basic mathematical concepts and demands spatial cognition during the learning process of shapes, size, quantity, and length (Biber et al., 2013; Hertanti et al., 2019). Previous studies have shown that spatial cognition is a good predictor of mathematics achievement, specifically geometry (Delgado and Prieto, 2004). Similarly, our results are in line with the idea that spatial cognition is relevant to geometry performance. Our analysis correlation between MRT and geometry problem-solving underscores the critical role that spatial cognition plays in mathematical achievement and has implications for educational practices.

There seems to be a lack of evidence for neural correlates of children’s cognitive effort and MRT, maybe for neuroimaging technique limitations on movement artifacts. However, the last few years have shown an increasing technology development that allows studies in schools with children (Soltanlou et al., 2018a), which includes naturalistic experimental settings with fNIRS (Ferrari and Quaresima, 2012; Balardin et al., 2017; Barreto et al., 2021) and Eye-Tracking (Epelboim and Suppes, 2001; Bolden et al., 2015) outside the laboratory environment. Our study is in line with the idea that fNIRS and eye-tracking are suitable for measuring the cognitive processes of schoolchildren in naturalistic settings (Mücke et al., 2018), and eye-tracking is a valuable device to use to measure physiological activations during spatial cognition stimuli in a familiar environment.

In addition to measuring cortical oxygenation, pupil diameter is also an important physiological measure of cognitive effort. Previous studies have suggested that changes in pupil size could be an index of how intensely the processing system is operating (Just and Carpenter, 1993; Laeng et al., 2012). The locus coeruleus (LC) is a brainstem nucleus that represents the primary source of norepinephrine (NE) in the brain (Kardon, 2005). The LC-NE system modulates the autonomic nervous system that influences pupil diameter through parasympathetic constriction and sympathetic dilation pathway (McDougal and Gamlin, 2008). The seminal study of Aston-Jones and Cohen (2005) demonstrated that pupil size is straightly related to fluctuations of the LC-NE activity. The same noradrenergic system has been implicated in arousal (Waterhouse, 2003; Aston-Jones, 2005; Aston-Jones et al., 2005) and mental effort (Bradley et al., 2008; Mathot, 2018). In fact, the LC widely projects throughout the PFC regions (Aston-Jones and Cohen, 2005) and seems to module neuronal network activity in cognitively demanding tasks (Robbins and Arnsten, 2009; Suttkus et al., 2021).

Although the LC-NE system presumably promotes the relationship between pupil dilation and mental effort, the mechanism in which the PFC activity areas influence the changes in pupil size is still not elucidated. Uncertainty about the mechanisms through the relationship between pupil size and PFC activity during cognitively demanding tasks emphasizes the need for further investigation into this matter. Multimodal studies have the potential to investigate how different areas of the nervous system are associated, revealing new levels of organization and functioning. For example, a recent multimodal ET-fNIRS study demonstrated a functional relationship between the lateral PFC and the LC that may vary at different difficulty levels during the work memory task (Yeung et al., 2021). It is already known that pupil dilates with cognitive load in adults (Van Der Meer et al., 2010; Van Der Wel and van Steenbergen, 2018) and children (McGarrigle et al., 2017). Our data relates schoolchildren’s pupil dilation during the MRT, suggesting a task-evoked increasing effort by spatial cognition demand. Further research should investigate the neural network specialization in visuospatial demand tasks across different age stages and neural development.

Interestingly, there are pieces of evidence that the brain activation of children can change after training. Soltanlou et al. (2018a) demonstrated that 2-week multiplication training with children reduced brain activation on the frontoparietal network. Furthermore, neuroimaging studies have suggested that children show decreased brain activation of the frontal cortex with increasing age (Houdé et al., 2010; Artemenko et al., 2018), which indicates reduced reliance on cognitive effort and attentional resources. Indeed, the cognitive effort is related to the degree of engagement with demanding tasks, and high engagement tends to enhance performance and attention (Kaplan and Berman, 2010). Considering the challenges to measuring the cognitive load (Paas et al., 2003), there is an increasing interest in the educational research field to apply a combination of measurements to obtain reliable and objective physiological data of cognitive effort in real-time (Kruger and Doherty, 2016).

The multimodal methodology has been used recently as a framework for the physiological measurement of cognitive load (Hosseini et al., 2017; Ís̨bilir et al., 2019; Larmuseau et al., 2020; Vanneste et al., 2021; Yeung et al., 2021). Studies applying pupillometry-fMRI concurrently measure have shown that pupil dilation was temporally correlated with activation of adults’ brain regions implicated in workload and cognitive control during working memory and decision-making tasks (Siegle et al., 2003; Satterthwaite et al., 2007). Similarly, we measured cortical activity and pupillary response concurrently using fNIRS and eye-tracking glasses to examine the neural systems linked to pupil dilation under mental rotation tasks. We also explored which PFC regions activate more during the visuospatial task. We believe that pupil dilations could be an index of mental effort differences indicating the mental effort linked to the allocation of cognitive resources on cognitively demanding tasks. We hypothesize that the change in pupil diameter could suggest how hard the student is trying to solve the problem, indicating the performance slightly. However, we did not apply different level difficulties in the MRT trials, which is a relevant limitation of our study. We found some short-channels signals significantly correlated to the task performance, which indicate extracerebral perfusion (e.g., forehead skin vessels) may possibly be an interference in our hemodynamic signals. Although such short channels do not correspond to long-distance channels that correlated significantly with the task performance, we considered this limitation worthy of mention.

One point of remark is that two fNIRS statistical analyses were conducted. The first focused on the significance of the contrast between task and rest. The second had as concern whether the amplitude of activation was correlated with behavioral data. We could use the results of the first analysis to select the channels for the second one. However, we opted to consider all channels in the latter and to be conservative by applying Bonferroni correction for multiple comparisons. We justify this choice since the two analyses are not only complementary, but they are testing different null hypotheses. The second point of remark is that although both oxy- and deoxy-Hb changes are consequences of the same neurophysiological process (the neurohemodynamic coupling process), the signal-to-noise ratio and susceptibility to systemic artifacts are not the same and may also depend on brain location. Thus, the statistical findings are not always reciprocal since the effect sizes are different. Moreover, for the same reasons, the lack of statistical power could explain why the correlation with task performance was only found at the right dlPFC.

## Conclusion

Our findings suggest that children with more mental effort during MRT performed better in the visuospatial and geometry problem-solving tests. The multimodal approach to monitoring students’ mental effort can be of great interest in providing objective feedback on cognitive resource conditions and advancing our comprehension of the neural mechanisms that underlie cognitive effort. Hence, the ability to detect two distinct mental states of rest or activation of children in real-time during the MRT could eventually lead to an application for investigating visuospatial skills of young students using naturalistic educational paradigms with a multimodal approach by applying the fNIRS and Eye-Tracker devices.

## Data availability statement

The raw data supporting the conclusions of this article will be made available by the authors, without undue reservation.

## Ethics statement

The studies involving human participants were reviewed and approved by the UFABC. Written informed consent to participate in this study was provided by the participants’ legal guardian/next of kin.

## Author contributions

RS and JR designed the study, collected and analyzed the data, and wrote the manuscript. AA and CB wrote and reviewed the manuscript. All authors have read and agreed to the published version of the manuscript.

## Footnotes

1
https://www.nitrc.org/projects/bnv

## References

[B1] Ahern S. Beatty J. (1979). Pupillary responses during information processing vary with Scholastic Aptitude Test scores. *Science* 205 1289–1292. 10.1126/science.472746 472746

[B2] Artemenko C. Soltanlou M. Ehlis A. C. Nuerk H. C. Dresler T. (2018). The neural correlates of mental arithmetic in adolescents: a longitudinal fNIRS study. *Behav. Brain Funct.* 14 1–13. 10.1186/s12993-018-0137-8 29524965 PMC5845230

[B3] Aston-Jones G. (2005). Brain structures and receptors involved in alertness. *Sleep medicine* 6 S3–S7. 16140243 10.1016/s1389-9457(05)80002-4

[B4] Aston-Jones G. Gonzalez M. M. Doran S. M. (2005). “Role of the locus coeruleus-norepinephrine system in arousal and circadian regulation of the sleep-waking cycle,” in *Norepinephrine: Neurobiology and Therapeutics for the 21st Century*, eds Ordway G. A. Schwartz M. Frazer A. (Cambridge, MA: Cambridge Univ. Press. in press).

[B5] Aston-Jones G. Cohen J. D. (2005). Adaptive gain and the role of the locus coeruleus–norepinephrine system in optimal performance. *J. Comp. Neurol.* 493 99–110. 16254995 10.1002/cne.20723

[B6] Ayaz H. Shewokis P. A. Bunce S. Izzetoglu K. Willems B. Onaral B. (2012a). Optical brain monitoring for operator training and mental workload assessment. *Neuroimage* 59 36–47. 10.1016/j.neuroimage.2011.06.023 21722738

[B7] Ayaz H. Shewokis P. A. Ízzetoğlu M. Çakır M. P. Onaral B. (2012b). “Tangram solved? Prefrontal cortex activation analysis during geometric problem solving,” in *2012 Annual International Conference of the IEEE Engineering in Medicine and Biology Society*, (IEEE), 4724–4727. 10.1109/EMBC.2012.6347022 23366983

[B8] Ayaz H. Willems B. Bunce B. Shewokis P. A. Izzetoglu K. Hah S. (2010). “Cognitive workload assessment of air traffic controllers using optical brain imaging sensors,” in *Advances in Understanding Human Performance: Neuroergonomics, Human Factors Design, and Special Populations*, eds Marek T. Karwowski W. Rice V. (Boca Raton: CRC Press Taylor & Francis Group), 21–31.

[B9] Balardin J. B. Zimeo Morais G. A. Furucho R. A. Trambaiolli L. Vanzella P. Biazoli Jr. (2017). Imaging brain function with functional near-infrared spectroscopy in unconstrained environments. *Front. Hum. Neurosci.* 11:258. 10.3389/fnhum.2017.00258 PMC5434677 28567011

[B10] Baltes P. B. Staudinger U. M. Lindenberger U. (1999). Lifespan psychology: theory and application to intellectual functioning. *Annu. Rev. Psychol.* 50 471–507. 10.1146/annurev.psych.50.1.471 15012462

[B11] Barreto C. de Albuquerque Bruneri G. Brockington G. Ayaz H. Sato J. R. (2021). A new statistical approach for fNIRS hyperscanning to predict brain activity of preschoolers’ using teacher’s. *Front. Hum. Neurosci.* 15:181. 10.3389/fnhum.2021.622146 34025373 PMC8137814

[B12] Bauer R. Jost L. Günther B. Jansen P. (2021a). Pupillometry as a measure of cognitive load in mental rotation tasks with abstract and embodied figures. *Psycholog. Res.* 2021 1–15. 10.1007/s00426-021-01568-5 34382111 PMC9177492

[B13] Bauer R. Jost L. Jansen P. (2021b). The effect of mindfulness and stereotype threat in mental rotation: a pupillometry study. *J. Cogn. Psychol.* 33 861–876.

[B14] Beatty J. Kahneman D. (1966). Pupillary changes in two memory tasks. *Psychon. Sci.* 5 371–372. 10.3758/BF03328444

[B15] Biber C. Tuna A. Korkmaz S. (2013). The mistakes and the misconceptions of the eighth grade students on the subject of angles. *Eur. J. Sci. Mathemat. Educ.* 1 50–59.

[B16] Boersma F. Wilton K. Barham R. Muir W. (1970). Effects of arithmetic problem difficulty on pupillary dilation in normals and educable retardates. *J. Exp. Child Psychol.* 9 142–155. 10.1016/0022-0965(70)90079-2 5452111

[B17] Bolden D. Barmby P. Raine S. Gardner M. (2015). How young children view mathematical representations: a study using eye-tracking technology. *Educ. Res.* 57 59–79.

[B18] Bradley M. M. Miccoli L. Escrig M. A. Lang P. J. (2008). The pupil as a measure of emotional arousal and autonomic activation. *Psychophysiology* 45 602–607. 18282202 10.1111/j.1469-8986.2008.00654.x PMC3612940

[B19] Bruce C. D. Hawes Z. (2015). The role of 2D and 3D mental rotation in mathematics for young children: what is it? Why does it matter? And what can we do about it? *ZDM Math. Educ.* 47 331–343. 10.1007/s11858-014-0637-4

[B20] Buckley J. Canty D. White D. Seery N. Campbell M. (2018). Spatial Working Memory and Neural Efficiency in Mental Rotations: an Insight from Pupillometry. *Eng. Design Graph. J.* 82:3.

[B21] Çakır M. P. Ayaz H. Ízzetoğlu M. Shewokis P. A. Ízzetoğlu K. Onaral B. (2011). Bridging brain and educational sciences: an optical brain imaging study of visuospatial reasoning. *Procedia-Soc. Behav. Sci.* 29 300–309.

[B22] Campbell M. J. Toth A. J. Brady N. (2018). Illuminating sex differences in mental rotation using pupillometry. *Biol. Psychol.* 138 19–26. 10.1016/j.biopsycho.2018.08.003 30086332

[B23] Carnoy M. Khavenson T. Fonseca I. Costa L. Marotta L. (2015). Is Brazilian education improving? Evidence from PISA and SAEB. *Cadernos de Pesquisa* 45 450–485.

[B24] Causse M. Chua Z. Peysakhovich V. Del Campo N. Matton N. (2017). Mental workload and neural efficiency quantified in the prefrontal cortex using fNIRS. *Sci. Rep.* 7 1–15. 10.1038/s41598-017-05378-x 28701789 PMC5507990

[B25] Chatham C. H. Frank M. J. Munakata Y. (2009). Pupillometric and behavioral markers of a developmental shift in the temporal dynamics of cognitive control. *Proc. Natl. Acad. Sci. U.S.A.* 106 5529–5533. 10.1073/pnas.0810002106 19321427 PMC2666994

[B26] Chevalier N. (2018). Willing to think hard? The subjective value of cognitive effort in children. *Child Dev.* 89 1283–1295. 10.1111/cdev.12805 28397991

[B27] Cohen M. S. Kosslyn S. M. Breiter H. C. DiGirolamo G. J. Thompson W. L. Anderson A. K. (1996). Changes in cortical activity during mental rotation a mapping study using functional MRI. *Brain* 119 89–100. 8624697 10.1093/brain/119.1.89

[B28] Curtin A. Ayaz H. (2018). The age of neuroergonomics: towards ubiquitous and continuous measurement of brain function with fNIRS. *Japan. Psycholog. Res.* 60 374–386.

[B29] Delgado A. R. Prieto G. (2004). Cognitive mediators and sex-related differences in mathematics. *Intelligence* 32 25–32. 10.1016/j.cognition.2018.10.005 30343180

[B30] Eckstein M. K. Guerra-Carrillo B. Singley A. T. M. Bunge S. A. (2017). Beyond eye gaze: What else can eyetracking reveal about cognition and cognitive development? *Developmental cognitive neuroscience* 25 69–91. 10.1016/j.dcn.2016.11.001 27908561 PMC6987826

[B31] Epelboim J. Suppes P. (2001). A model of eye movements and visual working memory during problem solving in geometry. *Vision Res.* 41 1561–1574. 10.1016/s0042-6989(00)00256-x 11343722

[B32] Ferrari M. Quaresima V. (2012). A brief review on the history of human functional near-infrared spectroscopy (fNIRS) development and fields of application. *Neuroimage* 63 921–935. 10.1016/j.neuroimage.2012.03.049 22510258

[B33] Fishburn F. A. Norr M. E. Medvedev A. V. Vaidya C. J. (2014). Sensitivity of fNIRS to cognitive state and load. *Front. Hum. Neurosci.* 8 1–11. 10.3389/fnhum.2014.00076 24600374 PMC3930096

[B34] Gao Y. Barreto A. Zhai J. (2007). “Digital filtering of pupil diameter variations for the detection of stress in computer users,” in *Proc 11th world multi-conference on systemics, cybernetics and informatics*, (Orlando).

[B35] Giofrè D. Mammarella I. C. Ronconi L. Cornoldi C. (2013). Visuospatial working memory in intuitive geometry, and in academic achievement in geometry. *Learn. Indiv. Diff.* 23 114–122.

[B36] Granholm E. Asarnow R. F. Sarkin A. J. Dykes K. L. (1996). Pupillary responses index cognitive resource limitations. *Psychophysiology* 33 457–461. 10.1111/j.1469-8986.1996.tb01071.x 8753946

[B37] Guillot A. Hoyek N. Collet C. (2012). Mental Rotation and Functional Learning. *Encyclopedia of the Sciences of Learning* 2012 2222–2223. 10.1007/978-1-4419-1428-6_493

[B38] Harris I. M. Miniussi C. (2003). Parietal lobe contribution to mental rotation demonstrated with rTMS. *J. Cogn. Neurosci.* 15 315–323. 10.1162/089892903321593054 12729485

[B39] Hertanti A. Retnawati H. Wutsqa D. U. (2019). The role of spatial experience in mental rotation. *J. Phys.* 1320:012043.

[B40] Hess E. H. Polt J. M. (1964). Pupil size in relation to mental activity during simple problem-solving. *Science* 143 1190–1192. 10.1126/science.143.3611.1190 17833905

[B41] Hess T. M. Ennis G. E. (2012). Age differences in the effort and costs associated with cognitive activity. *J. Gerontol. Series* 67 447–455. 10.1093/geronb/gbr129 PMC3391072 22131365

[B42] Hosseini S. M. Bruno J. L. Baker J. M. Gundran A. Harbott L. K. Gerdes J. C. (2017). Neural, physiological, and behavioral correlates of visuomotor cognitive load. *Sci. Rep.* 7 1–9. 10.1038/s41598-017-07897-z 28821719 PMC5562732

[B43] Houdé O. Rossi S. Lubin A. Joliot M. (2010). Mapping numerical processing, reading, and executive functions in the developing brain: an fMRI meta-analysis of 52 studies including 842 children. *Dev. Sci.* 13 876–885. 10.1111/j.1467-7687.2009.00938.x 20977558

[B44] Hugdahl K. Thomsen T. Ersland L. (2006). Sex differences in visuo-spatial processing: an fMRI study of mental rotation. *Neuropsychologia* 44 1575–1583. 10.1016/j.neuropsychologia.2006.01.026 16678867

[B45] Huppert T. J. (2016). Commentary on the statistical properties of noise and its implication on general linear models in functional near-infrared spectroscopy. *Neurophotonics* 3:010401. 10.1117/1.NPh.3.1.010401 26989756 PMC4773699

[B46] Hwang W. Y. Su J. H. Huang Y. M. Dong J. J. (2009). A study of multi-representation of geometry problem solving with virtual manipulatives and whiteboard system. *J. Educ. Technol. Soc.* 12 229–247.

[B47] Ís̨bilir E. Cakır M. P. Acartürk C. Tekerek A. S. (2019). Towards a multimodal model of cognitive workload through synchronous optical brain imaging and eye tracking measures. *Front. Hum. Neurosci.* 2019:375. 10.3389/fnhum.2019.00375 31708760 PMC6820355

[B48] Jainta S. Baccino T. (2010). Analyzing the pupil response due to increased cognitive demand: an independent component analysis study. *Internat. J. Psychophysiol.* 77 1–7. 10.1016/j.ijpsycho.2010.03.008 20381549

[B49] Johnson E. L. Miller Singley A. T. Peckham A. D. Johnson S. L. Bunge S. A. (2014). Task-evoked pupillometry provides a window into the development of short-term memory capacity. *Front. Psychol.* 5:218. 10.3389/fpsyg.2014.00218 24659980 PMC3952077

[B50] Jordan K. Heinze H. J. Lutz K. Kanowski M. Jäncke L. (2001). Cortical activations during the mental rotation of different visual objects. *Neuroimage* 13 143–152. 10.1006/nimg.2000.0677 11133317

[B51] Jurcak V. Tsuzuki D. Dan I. (2007). 10/20, 10/10, and 10/5 systems revisited: their validity as relative head-surface-based positioning systems. *Neuroimage* 34 1600–1611. 10.1016/j.neuroimage.2006.09.024 17207640

[B52] Just M. A. Carpenter P. A. (1993). The intensity dimension of thought: pupillometric indices of sentence processing. *Can. J. Exp. Psychol.* 47 310–339. 10.1037/h0078820 8364533

[B53] Kaplan S. Berman M. G. (2010). Directed Attention as a Common Resource for Executive Functioning and Self-Regulation. *Perspect. Psychol. Sci.* 5 43–57. 10.1177/1745691609356784 26162062

[B54] Karatekin C. (2004). Development of attentional allocation in the dual task paradigm. *Int. J. Psychophysiol.* 52 7–21. 10.1016/j.ijpsycho.2003.12.002 15003369

[B55] Karatekin C. Marcus D. J. Couperus J. W. (2007). Regulation of cognitive resources during sustained attention and working memory in 10-year-olds and adults. *Psychophysiology* 44 128–144. 10.1111/j.1469-8986.2006.00477.x 17241149

[B56] Kardon R. H. (2005). “Anatomy and physiology of the autonomic nervous system,” in *Wash and Hoyt’s Clinical Neuro-Ophthalmology*, 6th Edn, eds Miller N. R. Newman N. J. Biousse V. Kerrison J. B. (Philadelphia: Lippincott Williams & Wilkins), 649–714.

[B57] Khine M. S. (2016). “Spatial cognition: Key to STEM success,” in *Visual-spatial Ability in STEM Education: Transforming Research into Practice*, (New York, NY: Springer International Publishing), 3–8. 10.1007/978-3-319-44385-0_1

[B58] Klingner J. Tversky B. Hanrahan P. (2011). Effects of visual and verbal presentation on cognitive load in vigilance, memory, and arithmetic tasks. *Psychophysiology* 48 323–332. 10.1111/j.1469-8986.2010.01069.x 20718934

[B59] Koessler L. Maillard L. Benhadid A. Vignal J. P. Felblinger J. Vespignani H. (2009). Automated cortical projection of EEG sensors: anatomical correlation via the international 10–10 system. *Neuroimage* 46 64–72. 10.1016/j.neuroimage.2009.02.006 19233295

[B60] Kruger J. L. Doherty S. (2016). Measuring cognitive load in the presence of educational video: Towards a multimodal methodology. *Austral. J. Educ. Technol.* 32:6.

[B61] Kyttälä M. Lehto J. E. (2008). Some factors underlying mathematical performance: The role of visuospatial working memory and non-verbal intelligence. *Eur. J. Psychol. Educ.* 23 77–94. 10.1007/BF03173141

[B62] Laeng B. Sirois S. Gredebäck G. (2012). Pupillometry: a window to the preconscious? *Perspect. Psychol. Sci.* 7 18–27. 10.1177/1745691611427305 26168419

[B63] Larmuseau C. Cornelis J. Lancieri L. Desmet P. Depaepe F. (2020). Multimodal learning analytics to investigate cognitive load during online problem solving. *Br. J. Educ. Technol.* 51 1548–1562.

[B64] Lohman D. F. (1994). “Spatial ability,” in *Encyclopedia of intelligence*, ed. Sternberg R. J. (New York, NY: Macmillan), 1000–1007.

[B65] Lum J. A. Youssef G. J. Clark G. M. (2017). Using pupillometry to investigate sentence comprehension in children with and without specific language impairment. *J. Speech, Lang. Hear. Res.* 60 1648–1660. 10.1044/2017_JSLHR-L-16-0158 28586855

[B66] Mathot S. (2018). Pupillometry: psychology, physiology, and function. *J. Cogn.* 1:1. 10.5334/joc.18 PMC6634360 31517190

[B67] McCabe D. P. Roediger H. L. III McDaniel M. A. Balota D. A. Hambrick D. Z. (2010). The relationship between working memory capacity and executive functioning: evidence for a common executive attention construct. *Neuropsychology* 24:222. 10.1037/a0017619 20230116 PMC2852635

[B68] McDougal D. H. Gamlin P. D. R. (2008). “Pupillary control pathways,” in *The Senses: A Comprehensive Reference*, Vol. 1 eds Masland R. H. Albright T. (San Diego, CA: Academic Press), 521–536. 10.1016/B978-012370880-9.00282-6

[B69] McGarrigle R. Dawes P. Stewart A. J. Kuchinsky S. E. Munro K. J. (2017). Pupillometry reveals changes in physiological arousal during a sustained listening task. *Psychophysiology* 54 193–203. 10.1111/psyp.12772 27731503

[B70] Miller E. K. Cohen J. D. (2001). An integrative theory of prefrontal cortex function. *Annu. Rev. Neurosci.* 24 167–202. 11283309 10.1146/annurev.neuro.24.1.167

[B71] Mücke M. Andrä C. Gerber M. Pühse U. Ludyga S. (2018). Moderate-to-vigorous physical activity, executive functions and prefrontal brain oxygenation in children: a functional near-infrared spectroscopy study. *J. Sports Sci.* 36 630–636. 10.1080/02640414.2017.1326619 28538108

[B72] Orde B. J. (1996). *A correlational analysis of drawing ability and spatial ability.* Laramie, WY: University of Wyoming.

[B73] Paas F. G. Tuovinen J. E. Tabbers H. Van Gerven P. W. M. (2003). Cognitive load measurement as a means to advance cognitive load theory. *Educ. Psychol.* 38 63–71. 10.1207/S15326985EP3801_8 33486653

[B74] Pak R. (2001). “A further examination of the influence of spatial abilities on computer task performance in younger and older adults,” in *Proceedings of the Human Factors and Ergonomics Society Annual Meeting*, Vol. 45 (Los Angeles, CA: SAGE Publications), 1551–1555.

[B75] Prat S. C. Keller A. T. Just M. (2007). Individual differences in sentence comprehension: a functional magnetic resonance imaging investigation of syntactic and lexical processing demands. *J. Cogn. Neurosci.* 2007:66155197. 10.1184/R1/6615197.V1 PMC2599910 17892384

[B76] Robbins T. W. Arnsten A. F. T. (2009). The neuropsychopharmacology of fronto-executive function: monoaminergic modulation. *Annu. Rev. Neurosci.* 32 267–287. 10.1146/annurev.neuro.051508.135535 19555290 PMC2863127

[B77] Roberts J. E. Bell M. A. (2000). Sex differences on a mental rotation task: variations in electroencephalogram hemispheric activation between children and college students. *Dev. Neuropsychol.* 17 199–223. 10.1207/S15326942DN1702_04 10955203

[B78] Rojas-Líbano D. Wainstein G. Carrasco X. Aboitiz F. Crossley N. Ossandón T. (2019). A pupil size, eye-tracking and neuropsychological dataset from ADHD children during a cognitive task. *Scientific Data* 6 1–6. 10.1038/s41597-019-0037-2 30975993 PMC6472382

[B79] Rozado D. Andreas D. (2015). Combining EEG with pupillometry to improve cognitive workload detection. *Computer* 48 18–25.

[B80] SAEB (2011). *Geometry Problems from SAEB 2011 – INEP.* Avaiable online at: https://www.gov.br/inep/pt-br/areas-de-atuacao/avaliacao-e-exames-educacionais/saeb (accessed February 29, 2020).

[B81] Satterthwaite T. D. Green L. Myerson J. Parker J. Ramaratnam M. Buckner R. L. (2007). Dissociable but inter-related systems of cognitive control and rewardduring decision making: evidence from pupillometry and event-related fMRI. *NeuroImage* 37 1017–1031. 10.1016/j.neuroimage.2007.04.066 17632014

[B82] Shaw E. P. Rietschel J. C. Hendershot B. D. Pruziner A. L. Miller M. W. Hatfield B. D. (2018). Measurement of attentional reserve and mental effort for cognitive workload assessment under various task demands during dual-task walking. *Biolog. Psychol.* 134 39–51. 10.1016/j.biopsycho.2018.01.009 29378284

[B83] Shepard R. N. Metzler J. (1971). Mental rotation of three-dimensional objects. *Science* 171 701–703. 10.1126/science.171.3972.701 5540314

[B84] Shepard S. Metzler D. (1988). Mental Rotation: effects of Dimensionality of Objects and Type of Task. *J. Exp. Psychol. Hum. Percept. Perform.* 14 3–11. 10.1037/0096-1523.14.1.3 2964504

[B85] Shin J. Von Lühmann A. Kim D. W. Mehnert J. Hwang H. J. Müller K. R. (2018). Simultaneous acquisition of EEG and NIRS during cognitive tasks for an open access dataset. *Scient. Data* 5 1–16. 10.1038/sdata.2018.3 29437166 PMC5810421

[B86] Siegle G. J. Steinhauer S. R. Stenger V. A. Konecky R. Carter C. S. (2003). Use ofconcurrent pupil dilation assessment to inform interpretation and analysis offMRI data. *NeuroImage* 20 114–124. 10.1016/S1053-8119(03)00298-2 14527574

[B87] Sladky R. Stepniczka I. Boland E. Tik M. Lamm C. Hoffmann A. (2016). Neurobiological differences in mental rotation and instrument interpretation in airline pilots. *Sci. Rep.* 6 1–6. 10.1038/srep28104 27323913 PMC4914984

[B88] Soltanlou M. Artemenko C. Ehlis A. C. Huber S. Fallgatter A. J. Dresler T. (2018a). Reduction but no shift in brain activation after arithmetic learning in children: a simultaneous fNIRS-EEG study. *Sci. Rep.* 8 1–15. 10.1038/s41598-018-20007-x 29374271 PMC5786008

[B89] Soltanlou M. Sitnikova M. A. Nuerk H. C. Dresler T. (2018b). Applications of functional near-infrared spectroscopy (fNIRS) in studying cognitive development: the case of mathematics and language. *Front. Psychol.* 9:277. 10.3389/fpsyg.2018.00277 29666589 PMC5891614

[B90] Suttkus S. Schumann A. de la Cruz F. Bär K. J. (2021). Working memory in schizophrenia: The role of the locus coeruleus and its relation to functional brain networks. *Brain Behav.* 11:e02130. 10.1002/brb3.2130 33784023 PMC8119871

[B91] Sweller J. Chandler P. (1991). Evidence for cognitive load theory. *Cogn. Instruct.* 8 351–362.

[B92] Sweller J. van Merrienboer J. J. G. Paas F. G. W. C. (1998). Cognitive Architecture and Instructional Design. *Educ. Psychol. Rev.* 10 251–296. 10.1023/A:1022193728205

[B93] Tolar T. D. Lederberg A. R. Fletcher J. M. (2009). A structural model of algebra achievement: computational fluency and spatial visualisation as mediators of the effect of working memory on algebra achievement. *Educ. Psychol.* 29 239–266. 10.1080/01443410802708903

[B94] Toth A. J. Campbell M. J. (2019). Investigating sex differences, cognitive effort, strategy, and performance on a computerised version of the mental rotations test via eye tracking. *Sci. Rep.* 9 1–11. 10.1038/s41598-019-56041-6 31857671 PMC6923419

[B95] Tzuriel D. Egozi G. (2010). Gender differences in spatial ability of young children: the effects of training and processing strategies. *Child Dev.* 81 1417–1430. 10.1111/j.1467-8624.2010.01482.x 20840231

[B96] Van Der Meer E. Beyer R. Horn J. Foth M. Bornemann B. Ries J. (2010). Resource allocation and fluid intelligence: Insights from pupillometry. *Psychophysiology* 47 158–169. 10.1111/j.1469-8986.2009.00884.x 19761522

[B97] Van Der Wel P. van Steenbergen H. (2018). Pupil dilation as an index of effort in cognitive control tasks: a review. *Psychon. Bull. Rev.* 25 2005–2015. 10.3758/s13423-018-1432-y 29435963 PMC6267528

[B98] Vanneste P. Raes A. Morton J. Bombeke K. Van Acker B. B. Larmuseau C. (2021). Towards measuring cognitive load through multimodal physiological data. *Cogn. Technol. Work* 23 567–585.

[B99] Vassena E. Gerrits R. Demanet J. Verguts T. Siugzdaite R. (2019). Anticipation of a mentally effortful task recruits Dorsolateral Prefrontal Cortex: an fNIRS validation study. *Neuropsychologia* 123 106–115. 10.1016/j.neuropsychologia.2018.04.033 29705065

[B100] Vassena E. Silvetti M. Boehler C. N. Achten E. Fias W. Verguts T. (2014). Overlapping neural systems represent cognitive effort and reward anticipation. *PLoS One* 9:e91008. 10.1371/journal.pone.0091008 24608867 PMC3946624

[B101] Wai J. Lubinski D. Benbow C. P. (2009). Spatial ability for STEM domains: aligning over 50 years of cumulative psychological knowledge solidifies its importance. *J. Educ. Psychol.* 101 817–835. 10.1037/a0016127

[B102] Waterhouse B. D. (2003). The locus coeruleus-noradrenergic system: modulation of behavioral state and state-dependent cognitive processes. *Brain Res. Brain Res. Rev.* 42 33–84. 10.1016/s0165-0173(03)00143-7 12668290

[B103] Webb R. M. Lubinski D. Benbow C. P. (2007). Spatial ability: a neglected dimension in talent searches for intellectually precocious youth. *J. Educ. Psychol.* 99 397–420. 10.1037/0022-0663.99.2.397

[B104] Wei W. Yuan H. Chen C. Zhou X. (2012). Cognitive correlates of performance in advanced mathematics. *Br. J. Educ. Psychol.* 82 157–181. 10.1111/j.2044-8279.2011.02049.x 22429063

[B105] Westbrook A. Braver T. S. (2015). Cognitive effort: a neuroeconomic approach. *Cogn. Affect. Behav. Neurosci.* 15 395–415. 25673005 10.3758/s13415-015-0334-y PMC4445645

[B106] Westbrook A. Kester D. Braver T. S. (2013). What is the subjective cost of cognitive effort? load, trait, and aging effects revealed by economic preference. *PLoS One* 8:e68210. 10.1371/journal.pone.0068210 23894295 PMC3718823

[B107] Wu D. D. Yang J. F. Xie S. Luo J. T. Chang C. Q. Li H. (2020). An fNIRS examination of the neural correlates of mental rotation in preschoolers. *Hum. Behav. Brain* 1 37–42.

[B108] Xia M. Wang J. He Y. (2013). BrainNet viewer: a network visualization tool for human brain connectomics. *PLoS* O*ne* 8:e68910. 10.1371/journal.pone.0068910 23861951 PMC3701683

[B109] Yeung M. K. Lee T. L. Han Y. M. Chan A. S. (2021). Prefrontal activation and pupil dilation during n-back task performance: a combined fNIRS and pupillometry study. *Neuropsychologia* 159:107954. 10.1016/j.neuropsychologia.2021.107954 34252415

[B110] Zacks J. M. (2008). Neuroimaging studies of mental rotation: a meta-analysis and review. *J. Cogn. Neurosci.* 20 1–19. 10.1162/jocn.2008.20013 17919082

[B111] Zacks J. M. Gilliam F. Ojemann J. G. (2003). Selective disturbance of mental rotation by cortical stimulation. *Neuropsychologia* 41 1659–1667. 10.1016/S0028-3932(03)00099-X 12887990

