# Supplementary material for: Applying functional near-infrared spectroscopy and eye-tracking in a naturalistic educational environment to investigate physiological aspects that underlie the cognitive effort of children during mental rotation tests
Source: Front Hum Neurosci. 2022 Aug 12;16:889806. doi: 10.3389/fnhum.2022.889806 (PMC9442578; doi:10.3389/fnhum.2022.889806)
Supplement: Supplementary file 1 [file Data_Sheet_1.PDF]

Suplementary material 1

Channel	Label	Gyri(s)	Anatomical structures	BA
#1	Fpz- Fp1	M FL - L FL	Bilateral medial - Superior frontal gyrus	10
#2	Fpz- Afz	M FL	Bilateral medial	10 - 9
#3	Fpz-Fp2	M FL - R FL	Bilateral medial - Superior frontal gyrus	10
#5	Fp1-AF7	L FL	Superior frontal gyrus - Middle frontal gyrus	10
#6	AF7-F5	L FL	Middle frontal gyrus	10 - 46
#8	F5-F3	L FL	Middle frontal gyrus	46 - 8
#9	F3-F1	L FL	Middle frontal gyrus - Superior frontal gyrus	8 - 6
#11	F1-Fz	L FL - M FL	Superior frontal gyrus - Bilateral medial	6
#12	Afz-Fz	M FL	Bilateral medial	9 - 6
#13	Fz-F2	M FL - R FL	Bilateral medial - Superior frontal gyrus	6
#15	F2-F4	R FL	Superior frontal gyrus - Middle frontal gyrus	6 - 8
#16	F4-F6	R FL	Middle frontal gyrus	8 - 46
#18	AF4-Afz	R FL - M FL	Superior frontal gyrus - Bilateral medial	9
#19	F6-AF4	R FL	Middle frontal gyrus - Superior frontal gyrus	46 - 9
#20	Fp2-AF4	R FL	Superior frontal gyrus	10 - 9
#22	AF8-F6	R FL	Middle frontal gyrus	10 - 46
#23	AF8-Fp2	R FL	Middle frontal gyrus - Superior frontal gyrus	10
#25	Fp1-AF3	L FL	Superior frontal gyrus	10 - 9
#26	F5-AF3	L FL	Middle frontal gyrus - Superior frontal gyrus	46 - 9
#27	AF3-AFz	L FL - M FL	Superior frontal gyrus - Bilateral medial	9

Coordinates (EEG 10-10 system), anatomical structures and fNIRS channels. M: medial, R: right, L: left, FL: frontal lobe, #: channel, BA: Brodmann's area (Koessler et al., 2009).
