# Supplementary material for: Applying functional near-infrared spectroscopy and eye-tracking in a naturalistic educational environment to investigate physiological aspects that underlie the cognitive effort of children during mental rotation tests
Source: Front Hum Neurosci. 2022 Aug 12;16:889806. doi: 10.3389/fnhum.2022.889806 (PMC9442578; doi:10.3389/fnhum.2022.889806)
Supplement: Supplementary file 2 [file Data_Sheet_2.PDF]

Suplementary material 2

	MRT				GPS			
	Oxy-Hb		Deoxy-Hb		Oxy-Hb		Deoxy-Hb	
Channel	R	P (two tailed)	R	P (two tailed)	R	P (two tailed)	R	P (two tailed)
#1	0.318	0.080	0.029	0.876	0.235	0.201	0.018	0.924
#2	0.287	0.117	0.106	0.570	0.313	0.085	0.148	0.426
#3	0.247	0.180	0.090	0.627	0.232	0.208	0.335	0.065
"Ch.4	0.324	0.075	0.071	0.700	0.218	0.237	0.329	**0.007***
#5	0.409	**0.022***	0.228	0.217	0.296	0.106	0.344	0.058
#6	0.143	0.442	0.010	0.954	0.319	0.079	0.352	0.052
"Ch.7	0.388	**0.031***	-0.027	0.885	0.392	**0.029***	0.408	**0.022***
#8	-0.076	0.685	0.073	0.693	0.303	0.096	0.400	**0.025***
#9	-0.0376	0.840	0.299	0.101	0.112	0.547	0.207	0.263
"Ch.10	-0.0354	0.849	-0.112	0.549	0.140	0.449	0.245	0.182
#11	-0.098	0.598	0.232	0.208	0.005	0.977	-0.036	0.846
#12	0.041	0.822	-0.027	0.884	0.270	0.141	-0.110	0.555
#13	-0.220	0.233	0.023	0.900	-0.077	0.680	-0.121	0.516
"Ch.14	0.086	0.643	0.308	0.092	-0.068	0.714	0.072	0.699
#15	-0.164	0.377	0.299	0.102	-0.019	0.917	0.038	0.836
#16	0.035	0.851	-0.011	0.953	0.213	0.249	-0.0006	0.997
"Ch.17	0.042	0.820	0.157	0.397	0.152	0.412	-0.098	0.600
#18	0.017	0.925	-0.027	0.882	0.050	0.787	-0.059	0.752
#19	0.071	0.704	-0.102	0.584	0.058	0.753	0.007	0.968
#20	0.150	0.420	-0.144	0.439	0.178	0.337	0.153	0.410
"Ch.21	0.117	0.530	0.304	0.096	0.203	0.272	0.283	0.122
#22	-0.006	0.972	-0.109	0.559	0.176	0.342	0.191	0.301
#23	0.088	0.637	-0.128	0.493	0.233	0.205	0.312	0.087
"Ch.24	0.348	0.055	-0.077	0.677	0.168	0.364	0.397	**0.026***
#25	0.446	**0.011***	-0.041	0.826	0.205	0.268	0.284	0.121
#26	0.089	0.630	0.072	0.699	0.144	0.439	0.347	0.055
#27	0.246	0.180	-0.249	0.894	-0.011	0.952	0.124	0.506
"Ch.28	0.167	0.369	0.041	0.828	-0.042	0.820	0.229	0.215

Correlation between visuospatial test scores and beta values extracted after SPM analyses. MRT: mental rotation task, GPS: geometry problem-solving, Oxy-Hb: oxyhemoglobin, Deoxy-Hb: deoxyhemoglobin, R: Spearman correlation, #: Channel, “Ch.: short-distance channel, * p<0.05.
