# Supplementary material for: Applying functional near-infrared spectroscopy and eye-tracking in a naturalistic educational environment to investigate physiological aspects that underlie the cognitive effort of children during mental rotation tests
Source: Front Hum Neurosci. 2022 Aug 12;16:889806. doi: 10.3389/fnhum.2022.889806 (PMC9442578; doi:10.3389/fnhum.2022.889806)
Supplement: Supplementary file 3 [file Data_Sheet_3.PDF]

Suplementary material 3

	Oxy-Hb			Deoxy-Hb		
**Channel**	**T test**	**p-value**	**p-corrected**	**T test**	**p-value**	**p-corrected**
#1	-0.937	0.822	1	1.287	0.104	1
#2	-0.919	0.817	1	1.201	0.119	1
#3	1.039	0.153	1	0.873	0.195	1
"Ch.4	1.172	0.125	1	2.622	**0.007***	0.134
#5	0.721	0.238	1	2.645	**0.006***	0.127
#6	2.407	**0.011***	0.222	4.074	**<0.001***	**0.002**
"Ch.7	1.299	0.102	1	3.054	**0.002***	0.046
#8	-2.005	0.973	1	1.157	0.128	1
#9	-2.975	0.997	1	-0.312	0.622	1
"Ch.10	-2.639	0.994	1	-0.914	0.816	1
#11	-3.125	0.998	1	1.341	0.095	1
#12	-1.743	0.954	1	-1.513	0.930	1
#13	-1.540	0.933	1	-0.261	0.602	1
"Ch.14	-4.372	1.000	1	1.248	0.111	1
#15	-2.717	0.995	1	2.431	**0.011***	0.210
#16	0.005	0.498	1	2.542	**0.008***	0.162
"Ch.17	-1.915	0.968	1	0.925	0.181	1
#18	-2.691	0.994	1	0.180	0.429	1
#19	0.844	0.202	1	3.261	**0.001***	0.027
#20	-0.189	0.574	1	1.821	**0.039***	0.783
"Ch.21	-0.522	0.697	1	1.352	0.093	1
#22	1.770	**0.043***	0.866	4.254	**<0.001***	**0.001**
#23	3.015	**0.003***	0.05	5.046	**<0.001***	**<0.001**
“Ch.24	0.699	0.245	1	2.045	**0.025***	0.493
#25	-1.137	0.868	1	0.666	0.255	1
#26	-0.351	0.636	1	2.073	**0.023***	0.465
#27	-3.127	0.998	1	0.200	0.421	1
"Ch.28	-2.647	0.994	1	0.751	0.229	1

Statistically significant test of the beta values for hemodynamic concentration changes compared with the baseline rest condition on each channel. Bold font indicates statistical significance *p-value <0.05 and Bonferroni-corrected p-value (according to 20 long-channels) on T-test. Oxy-Hb: oxyhemoglobin, Deoxy-Hb: deoxyhemoglobin, #: channel, “Ch.: short-distance channel.
