# Supplementary figures and images for: Applying functional near-infrared spectroscopy and eye-tracking in a naturalistic educational environment to investigate physiological aspects that underlie the cognitive effort of children during mental rotation tests
Source: Front Hum Neurosci. 2022 Aug 12;16:889806. doi: 10.3389/fnhum.2022.889806 (PMC9442578; doi:10.3389/fnhum.2022.889806)

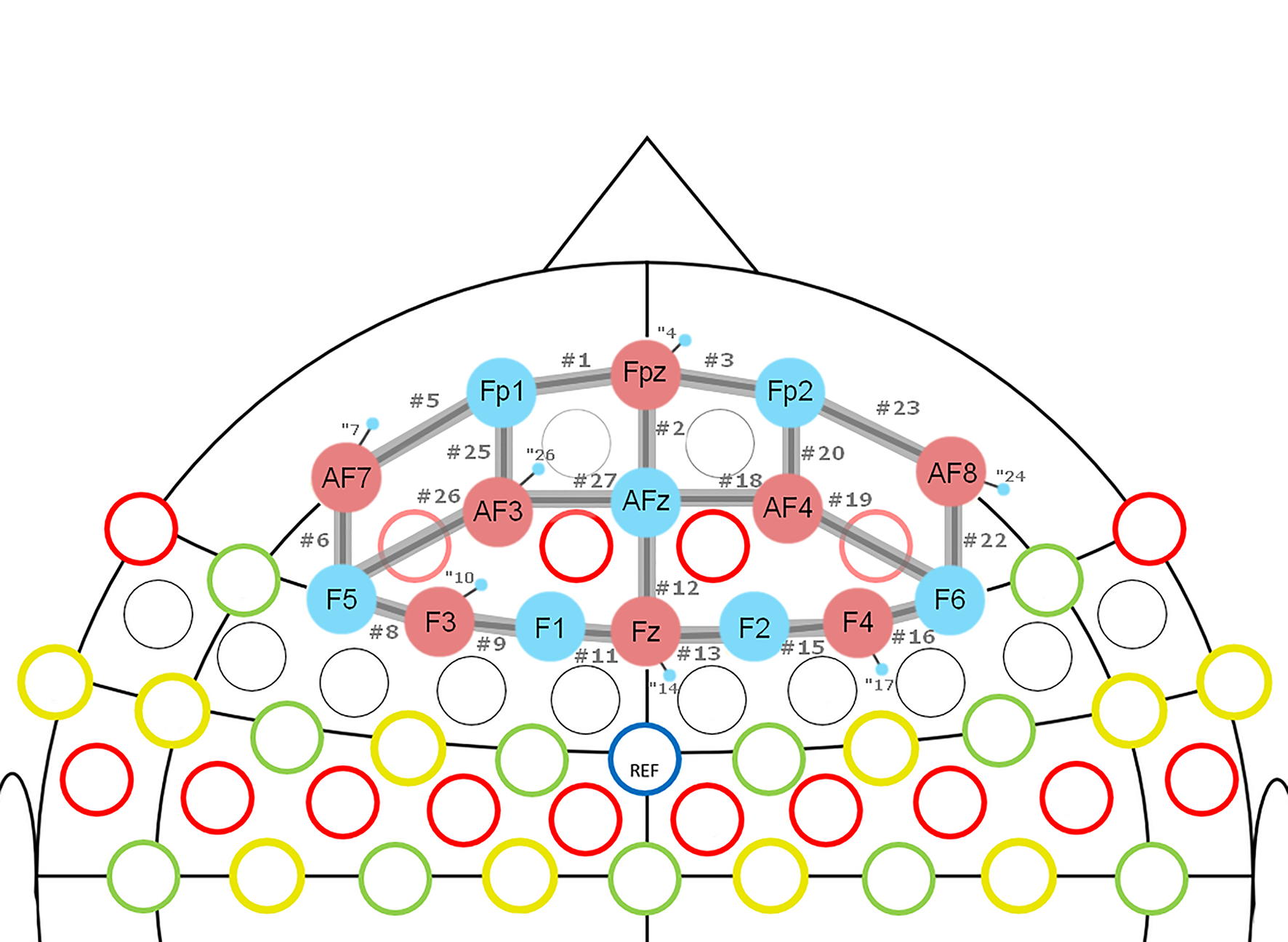

Supplement: Supplementary file 4 [file Image_1.TIF]
